# Non-surgical orbital decompression using diuresis in dysthyroid optic neuropathy: a case report

**DOI:** 10.1530/ETJ-22-0078

**Published:** 2022-07-03

**Authors:** Annamaria Erdei, Annamaria Gazdag, Bernadett Ujhelyi, Edit B Nagy, Ervin Berenyi, Eszter Berta, Zita Steiber, Sandor Barna, Emese Mezosi, Miklos Bodor, Endre V Nagy

**Affiliations:** 1 Division of Endocrinology, Department of Internal Medicine, Faculty of Medicine, University of Debrecen, Debrecen, Hungary; 2 Department of Ophthalmology, Faculty of Medicine, University of Debrecen, Debrecen, Hungary; 3 Division of Radiology and Imaging Science, Department of Medical Imaging, Faculty of Medicine, University of Debrecen, Debrecen, Hungary; 4 Division of Nuclear Medicine, Department of Medical Imaging, Faculty of Medicine, University of Debrecen, Debrecen, Hungary; 5 First Department of Internal Medicine, University of Pecs Medical School, Pecs, Hungary

**Keywords:** thyroid eye disease, dysthyroid optic neuropathy, diuretics, MRI

## Abstract

**Introduction:**

Dysthyroid optic neuropathy (DON) is a rare, severe form of thyroid eye disease, in which decreased visual acuity is accompanied by characteristic MRI findings. The treatment of DON has always been a challenge.

**Case presentation:**

In a patient in whom visual acuity deteriorated on the left eye, mannitol 20% 200 mL followed by furosemide 40 mg 6 h later, administered daily, were initiated on the day of admission. Visual function by ophthalmology methods, and orbital compartment volumes and water content by MRI were followed. Intravenous diuretics resulted in an immediate therapeutic response. Visual acuity improved from 20/50 to 20/25 after 2 days of treatment. MRI revealed decreasing water content of both the muscle and connective tissue compartments without any volume changes. Subsequently, corticosteroids and orbital irradiation were started. Orbital decompression surgery was not required.

**Discussion/conclusion:**

Edematous swelling of orbital tissues is an established contributor of local pressure increase in thyroid eye disease. Diuretics reduce orbital pressure and, if confirmed by others, may be useful additions to the standard of care in sight-threatening DON.

**Established facts**

- For dysthyroid optic neuropathy, high-dose i.v. corticosteroids are the standard therapy. If visual acuity fails to improve, orbital decompression surgery has to be considered.

**Novel insights:**

- Starting the treatment with diuretics alone resulted in immediate improvement of visual acuity. As evidenced by MRI, the mechanism is a decrease in the water content of orbital tissues. These findings suggest that the addition of diuretics to the standard of care might help to escape orbital surgery in dysthyroid optic neuropathy.

## Introduction

Thyroid eye disease (TED) is an autoimmune inflammatory disease affecting the orbital connective tissue and extraocular muscles which represents a major challenge during the treatment of Graves’ disease. Sight-threatening compressive dysthyroid optic neuropathy (DON) is the most severe complication of TED. The main clinical finding is the progressive loss of visual acuity, which, if not treated appropriately, may result in the complete loss of vision. High-dose i.v. methylprednisolone pulses are the standard therapy for sight-threatening DON (1). If the response to conservative treatment is absent or poor, that is, visual acuity fails to improve within a few days, surgical orbital decompression has to be performed without delay. Resection of the bony walls of the orbit is the usual approach, which may be supplemented with connective tissue removal (2).

Diuretics are an established treatment for cerebral edema and may have the potential to reduce the edematous swelling of orbital tissues. To clarify if diuresis is beneficial, in a patient with DON, corticosteroids were delayed and for the first 3 days only diuretics were administered. As of now, there have been no published cases on the therapeutic potential of diuretics in the management of DON.

## Case report

A 69-year-old man was referred to our Thyroid Eye Clinic for progressive TED. Graves’ hyperthyroidism and atrial fibrillation had been diagnosed 8 months earlier. He had become euthyroid on methimazole 10 mg per day; additional medications were propranolol, apixaban, and ramipril for pre-existing essential hypertension. He recalled that eyelid swelling, double vision, and excessive tearing were present for the last 4 months.

On presentation, his main complaint was progressive sight loss on the left eye over the last 2 weeks. General physical examination was unremarkable except irregular pulse of 80 bpm; symmetric exophthalmos and symmetric periorbital edema were seen. Low thyroid-stimulating hormone (TSH) (0.006 mU/L, reference range: 0.3-4.2 mU/L) was accompanied by normal free thyroxine and free triiodothyronine levels and elevated TSH receptor antibodies (TRAb: 10.9 U/L; reference range: <1.0 U/L).

Visual acuities were 20/20 on the right and 20/50 on the left eye. By slit-lamp examination, it was found that classical TED signs, periorbital and eyelid edema, exophthalmos, conjunctival hyperemia, chemosis, and swollen caruncles were present. No signs of corneal involvement were seen; the corneal epithelium was intact on both eyes with no fluorescein staining. Pupillary reactions were intact, direct and consensual on both eyes. Proptosis by Hertel exophthalmometry was 25 mm and 27 mm right and left, respectively. By kinetic perimetry, isopters were normal on the right eye, while concentric visual field angle reduction was observed on the left eye, especially in the temporal region (30°). Color visual fields were intact on the right eye; however, on the left eye, visual field and both red and green isopters were affected, and central color vision was deteriorated. By ophthalmoscopy, the optic nerve head did not show papillary edema on either side. No signs of optic nerve atrophy were present. The vascular structures showed mild signs of arteriosclerosis and hypertension. Critical flicker-fusion frequencies (CFF) were 34 Hz on the right and 29 Hz on the left (Table 1). CFF is the frequency at which flickering light can be perceived as continuous; values below 36 Hz are diagnostic of optic nerve or visual cortex dysfunction if there is no anterior segment pathology, lens opacification, vitreous body abnormality, or retinal disease. Clinical activity scores (CAS) were 7 and 8, right and left, respectively, on the 10-item CAS scale (3). Orbital MRI showed typical MRI features of TED with apical crowding on both sides. The diagnosis was sight-threatening DON.

**Table 1:** Response to diuretics.

	Visual acuity		CFF (Hz)		Proptosis (mm)		IOP (mmHg)		CAS		Volumes, left orbit (cm^3^)		Signal intensity ratio, left orbit	
	R	L	R	L	R	L	R	L	R	L	Muscle	Connec	Muscle	Connec
Day 1	20/20	20/50	34	29	25	27	18	18	7	8	6.93	23.83	18.31	2.14
Day 2	20/20	20/50	32	37	-	-	18	18	-	-	-	-	-	-
Day 3	20/20	20/25	36	36	-	-	17	20	-	-	-	-	-	-
Day 4	20/20	20/25	38	38	25	27	18	24	-	-	6.78	23.42	17.09	1.78
Day 8	20/20	20/25	36	38	25	27	17	22	6	7	-	-	-	-
Day 120	20/20	20/25	38	37	23	25	17	18	2	5	3.39	22.24	6.83	1.04

–, not done; CAS, clinical activity score (ten-point scale); CFF, critical flicker-fusion frequency; connec, connective tissue; IOP, intraocular pressure; L, left side; R, right side.

MRI of the orbits has been performed in a conventional 3T MRI unit (Philips Achieva 3.0T TX, Philips Healthcare, Amsterdam, the Netherlands). Axial non-contrast 3D T1-weighted gradient echo (TR 20 ms, TE 2 ms, slice thickness 2 mm) and short tau inversion recovery (STIR) (TR 4100 ms, TE 80 ms, IT 200 ms, slice thickness 3 mm) images were acquired. The extraocular muscles, optic nerve, intraorbital parts of the globe, and the lacrimal gland were manually segmented (4), and volumes were calculated using InterView Fusion 3.08.009 (Mediso Budapest, Hungary). These volumes were extracted from the total orbital volume to obtain connective tissue volume.

Signal intensity on STIR images as indicator of tissue edema (5) was measured in the coronal slice 10 mm behind the globe. Signal intensities of the full cross-sectional muscle area (including all extraocular muscles) and the entire connective tissue area were measured; mean intensities were calculated by the software package provided by the manufacturer of the MRI unit. Signal intensity ratio (SIR) was calculated (5) dividing the signal intensity of each compartment with that of the ipsilateral buccal fat pad.

As salvage therapy, mannitol 20% 200 mL was administered daily intravenously for 4 consecutive days (days 1 through 4). Each mannitol infusion was followed by furosemide 40 mg 6 h later. Corticosteroids were started on day 4; 500 mg methylprednisolone was administered on alternate days (day 4, day 6, and day 8), followed by 500 mg methylprednisolone weekly for 3 weeks and 250 mg methylprednisolone for 6 weeks. The protocol was approved by the Regional and Institutional Ethics Committee of the University of Debrecen. Consent was obtained from the patient after a full explanation of the purpose and nature of all procedures used.

**Figure 1:**
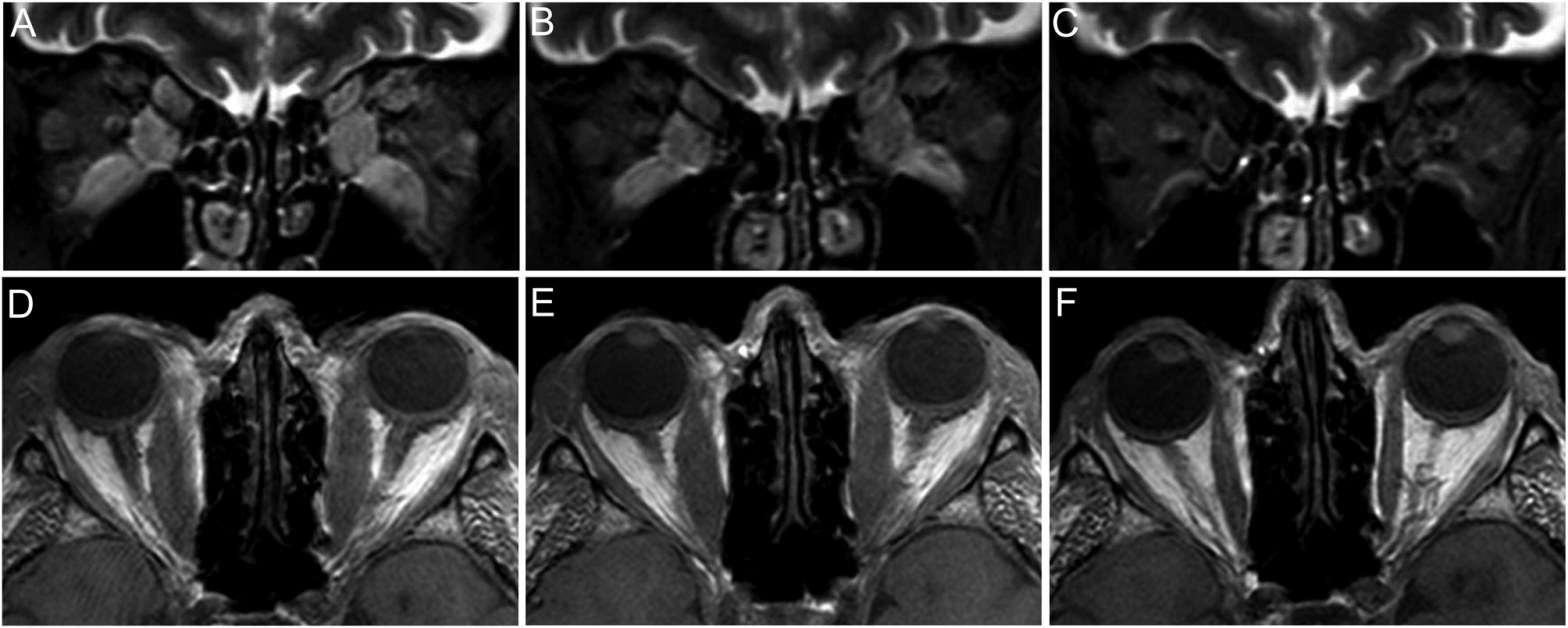
Treatment response. Short tau inversion recovery (STIR) coronal (A, B, and C) and T1-weighted axial (D, E, and F) MRI images. Higher signal intensities (bright areas) in the STIR images mean higher water content. (A and D) Day 1, before the administration of diuretics. (B and E) Day 4, after 3 days of diuretic treatment, before the first corticosteroid infusion. (C and F) Day 120, after diuretics and full course of corticosteroids combined with orbital irradiation.

Immediate improvement of the CFF on day 2 was followed by improvement of visual acuity on day 3 (Table 1). Improvement was observed in the visual field of the left eye. These changes were accompanied neither by muscle nor by connective tissue volume decrease on MRI (Fig. 1 and Table 1); however, a clear drop in SIR of both the muscle and connective tissue compartments reflected the decrease of the water content of these tissues (Table 1).

Improved visual acuity remained stable, and orbital decompression surgery was escaped. During the later course of the disease, orbital radiotherapy combined with i.v. glucocorticoids was effective (Fig. 1C and F). TSH and thyroid hormones were in the normal range during follow-up and TRAb decreased to near-normal (1.2 U/L) by day 120.

## Discussion

Based on current guidelines (1), the suggested therapeutic approach to DON is high-dose i.v. corticosteroids which may result in complete visual recovery in 43% of cases (6). If there is no improvement in visual acuity, orbital bony decompression follows (7), which results in permanent benefit in nearly all cases (8).

Edematous swelling of orbital tissues is a recognized phenomenon in active TED; water content estimated by MRI has been shown to be a corollary of disease activity (9). To clarify the potential benefit of diuretic treatment in a patient with sight-threatening DON, corticosteroids were delayed and diuretics alone were administered for 3 days; subsequently, corticosteroid was added starting on day 4. Optic nerve function was followed daily. The diuretic regime had an immediate effect; visual acuity improved within 2 days. MRI follow-up was included in the protocol; the day 4 MRI and ophthalmology examinations were performed after 3 days of diuretics but before the first corticosteroid infusion was administered on day 4. Albeit no reduction in the muscle and connective tissue volumes were seen by MRI on day 4, water content of these compartments was reduced as evidenced by the decrease of SIR. The decrease of edema of orbital tissues paralleled with the improvement of visual acuity. We interpret these changes as indirect signs of decreasing orbital pressure. We assume that minor orbital volume changes which remain undetectable by both MRI and Hertel’s exophthalmometry may result in substantial orbital pressure relief with an improvement of optic nerve function. Four months later, the volume of both orbital compartments was reduced, a known combined effect of the subsequent corticosteroid course and orbital irradiation.

As no corticosteroids (only diuretics) were administered between day 1 and day 4 ophthalmology evaluations, the improvement seen on day 4 may entirely be attributed to the orbital effect of diuretics. Mannitol is an osmotic diuretic used for rapid volume depletion and water deprivation in focal brain diseases. The high hyaluronan content of the orbital tissues could be similarly deprived of a certain amount of water; hyaluronan retains a large amount of water, approximately 700 times its own weight (10). Indeed, we found a measurable reduction of the water content of orbital tissues after mannitol administration. In the case presented here, we used diuretics and corticosteroids sequentially with no overlap. However, it is tempting to hypothesize that combined administration of mannitol with the first dose of either corticosteroid or teprotumumab (11) may bridge the gap during the first few days after starting the therapy, immediately easing optic nerve compression and allowing time for the development of the effect of the well-established treatment modalities.

In addition to being a single case report, another limitation of our work is that there is a mild chance that not the treatment but the natural course of TED, that is, coincidence with fluctuation in the disease activity, was responsible for the improvement. However, the course and timing of the changes argue against this. We did not experience any side effect of the diuretics, which, especially when co-administered with corticosteroids, may be of concern.

Administration of mannitol resulted in immediate improvement of visual acuity. It seems feasible to add mannitol to i.v. corticosteroids in DON. The percentage of patients who may avoid decompression surgery by the combined use of diuretics and corticosteroids remains to be elucidated.

## Declaration of interest

The authors declare that there is no conflict of interest that could be perceived as prejudicing the impartiality of this case report.

## Funding

This work was supported by the Hungarian National Research, Development and Innovation Office Grants NKFIH K116419 and UNKP-21-4.2.

## Study approval statement

The protocol was reviewed and approved by the Regional and Institutional Ethics Committee of the University of Debrecen, approval number 5952.

## Consent to publish statement

Written consent was obtained from the patient after full explanation of the purpose and nature of all procedures used, for publication of the details of his medical case and accompanying images.

## Data availability statement

All data related to this study will be made available on request by the corresponding author.

## Author contribution statement

Annamaria Erdei – diagnostic workup of the patient, treatment, follow-up, preparation of the mansucript. Annamaria Gazdag – treatment, follow-up, preparation of the manuscript. Bernadett Ujhelyi – ophthalmology examinations, preparation of the manuscript. Edit B Nagy – MRI examinations, preparation of the manuscript. Ervin Berenyi – MRI examinations, review of the manuscript. Eszter Berta – patient follow-up, literature review, manuscript preparation. Zita Steiber – ophthalmology examinations. Sandor Barna – patient follow-up, data management, literature review, manuscript preparation. Emese Mezosi – participated in the development of the new treatment modality. Miklos Bodor – patient follow-up, literature review, manuscript preparation. Endre V Nagy – development of the new treatment modality, study design, finalization and approval of the manuscript.
